# Editorial: Application of the Third Generation of Cognitive-Behavioral Approaches to Parenting

**DOI:** 10.3389/fpsyg.2019.02207

**Published:** 2019-09-27

**Authors:** Helena Moreira, Eva Potharst, Maria Cristina Canavarro

**Affiliations:** ^1^Center for Research in Neuropsychology and Cognitive-Behavioral Intervention, University of Coimbra, Coimbra, Portugal; ^2^UvA Minds, Academic Outpatient (Child and Adolescent) Treatment Center, University of Amsterdam, Amsterdam, Netherlands

**Keywords:** parenting, third generation of cognitive-behavioral therapy, mindfulness, compassion, acceptance

In recent years, there has been growing interest in understanding how the third generation of cognitive-behavioral approaches, particularly mindfulness- and acceptance-based approaches, can contribute to the design of more efficacious parenting interventions (Bögels et al., 2014; Coatsworth et al., 2014; Kirby, 2016; Whittingham and Coyne, 2019) and to a better understanding of parenting behaviors and the parent-child relationship (Brassell et al., 2016; Parent et al., 2016). For instance, it has been proposed that bringing mindful attention to parent-child interactions may improve the quality of parenting (Gouveia et al., 2016; Potharst et al., 2018), foster a more positive parent-child relationship (Medeiros et al., 2016; Chaplin et al., 2018), and promote better psychological functioning in children and their parents (Meppelink et al., 2016; Parent et al., 2016; Turpyn and Chaplin, 2016). Other psychological processes, such as (self-)compassion and acceptance, have also shown to play an important role in the way parents think and feel about parenting and in the way they interact with and relate to their children (Moreira et al., 2015; Brassell et al., 2016; Kirby, 2016; Whittingham et al., 2019).

However, the application of third-generation cognitive-behavioral therapies and concepts to parenting is still in its infancy, and further research is needed to explore the potential of these approaches to enhance existing parenting interventions or to inform the development of new parenting interventions targeting different groups of parents and their children. More research is also needed to understand how mindfulness, (self-)compassion, acceptance, and other related psychological processes may influence parenting practices, the parent-child relationship, and the child's socioemotional development.

In the current Research Topic, we brought together several researchers from different countries (Australia, Brazil, China, United States, Italy, Germany, Portugal, The Netherlands, and UK) that focused on the application of third-generation cognitive-behavioral therapies and models to parenting. Of the 14 articles published on this topic, five focused on exploring variables (e.g., experiential avoidance, compassion) that may influence parental behavior and their child's psychological functioning. The remaining studies focused on empirically-based parenting interventions informed by third-generation cognitive-behavioral therapies. These interventions were delivered in different formats (e.g., group and online) and were designed for different phases of the life cycle (e.g., transition to parenthood) and for different target groups (e.g., mothers of children with some developmental condition).

Two studies explored how parent factors, such as goal motivation, psychopathology, and work-family conflict, can influence parenting styles and behaviors. Kirby et al. showed that mothers' compassionate goals contributed to facilitative parenting, whereas self-focused goals were associated with greater psychologically controlling parenting. Moreira et al. found that mothers who experienced increased levels of work-family conflict were less mindful in their relationship with their children because they experienced higher levels of parenting stress and anxiety and depressive symptoms.

Two studies investigated the role of a mindful parenting style on the child's socioemotional functioning. Gouveia et al. found that higher levels of mindful parenting skills were associated with lower levels of emotional eating in adolescents. These authors also found that this association was mediated by the adolescents' self-compassion and body shame. In a study focusing on the association between mindful parenting and preschool children's decision-making behavior, Wong et al. found that higher levels of maternal mindful parenting predicted child prosocial decision-making behavior during a sharing task.

In another study focusing on the effect of parenting behaviors on child functioning, Emerson et al. examined the intergenerational relationship between parent and child anxiety and highlighted the key role of parental experiential avoidance. These authors found that parental control and parental experiential avoidance mediated the association between parent and child anxiety, and they also found that the association between parental control and child anxiety was only significant under conditions of high parental experiential avoidance.

The remaining studies focused on parenting interventions and included empirical studies testing the effects of those interventions, a systematic review and meta-analysis, a conceptual study, a study protocol, and an opinion article. Parent training programs that are either mindfulness-based or include mindfulness meditation are adjusted for specific populations of parents. Zeegers et al. focused on mothers with an infant- or toddler-aged child who experienced parenting stress and/or (co)regulatory difficulties. They showed that the mindful parenting group training, Mindful with your Baby/Toddler, was effective in improving parental stress, observed parental acceptance of the child and attunement to the child's mental world. Zhang et al. carried out an RCT in military families post-deployment, offering the mothers either services as usual or a parent training program that included mindfulness meditation in each of the 14 sessions (After Deployment Adaptive Parenting Tools program). They found that mothers with low trait mindfulness at baseline showed improved trait mindfulness at 1-year follow-up, and this mediated improvements in self-reported parenting skills at 2-year follow-up. Singh et al. offered a parent training program that included both mindfulness and positive behavior support (the Mindfulness-Based Positive Support Program) to parents of adolescents with either autism spectrum disorder or intellectual disabilities. In both of these groups, not only was a reduction of parental stress shown but also a reduction in adolescent aggression and an improvement in adolescent compliance behavior.

Psychological interventions, including mindfulness-based and other third generation cognitive-behavioral interventions, are increasingly offered via the internet (Spijkerman et al., 2016). Two studies in this Research Topic examined the effectiveness of online, self-directed interventions. Fonseca et al. offered a cognitive-behavioral intervention including elements of self-compassion and acceptance (Be a Mom) to women in the postpartum period who were at risk for postpartum depression. In an RCT, Be a Mom was compared with a waiting list and was shown to be effective in reducing symptoms of depression and emotion regulation difficulties and increasing self-compassion. Potharst et al. carried out an RCT in women with a toddler who were experiencing elevated levels of parental stress to whom they offered an online mindful parenting training. In comparison to the waiting list, the training was effective in improving self-compassion and in decreasing symptoms of anxiety and depression and parental over reactivity.

In a systematic review and meta-analysis, Burgdorf et al. assessed the effectiveness of mindfulness interventions for parents in reducing parenting stress and improving youth psychological outcomes. These authors concluded that mindfulness-based parenting interventions may reduce parenting stress and improve youth psychological functioning.

In three manuscripts on this Research Topic, new applications of cognitive-behavioral interventions for parents are presented. Cousineau et al. elaborate on the possibility and importance of offering training not only in mindfulness but also in compassion to parents with children with chronic disease or disability to alleviate parental burden and support child well-being; they introduce their Model of Compassion, Mindfulness, and Resilience in Parental Caregiving, on the basis of which a new intervention can be developed. In their manuscript presenting a research protocol, Lo et al. introduce the idea of enriching psychoeducation to families with an adult-aged child with psychosis with mindfulness. Because of the high levels of stress that these parents experience and the importance of family functioning in the prevention of relapse, family psychoeducation is included in treatment guidelines. In an RCT, they will study the added value of mindfulness for both the parents and young adults by comparing Mindfulness-Based Family Psychoeducation with regular family psychoeducation. The starting point for the manuscript by Grecucci et al. is the importance of internalized dysfunctional attachment, which, in parents, can take the form of mental representations of the child and the self as a parent. These lead to maladaptive coping strategies that negatively influence parenting and the parent-child relationship. The authors offer a two-step approach, in which mindfulness and acceptance techniques may be important and valuable additions to Schema Therapy in treating these mental representations because they may be supportive in containing the emotional experience associated with the mental representations, decreasing the acting out of the maladaptive coping strategies, and choosing new behavior that is values-based.

The manuscripts in this Research Topic have illustrated the importance of mindfulness and related psychological processes in parenting, the parent-child relationship and child development. The manuscripts showed the possibilities of third generation cognitive-behavioral approaches for a wide range of difficulties that families may encounter. The wide variety of application areas that were described in this Research Topic may, on the one hand, support and motivate researchers and clinicians to continue to adjust existing programs for specific target groups, but on the other hand, to return to the essence of these approaches, namely, to recognize common humanity and universal suffering in specific difficulties and the need for mindful acceptance and compassion.

## Author Contributions

All authors listed have made a substantial, direct and intellectual contribution to the work, and approved it for publication.

### Conflict of Interest

The authors declare that the research was conducted in the absence of any commercial or financial relationships that could be construed as a potential conflict of interest.
